# Persuading Others to Avoid Persuasion: Inoculation Theory and Resistant Health Attitudes

**DOI:** 10.3389/fpsyg.2016.00122

**Published:** 2016-02-09

**Authors:** Josh Compton, Ben Jackson, James A. Dimmock

**Affiliations:** ^1^Institute for Writing and Rhetoric, Dartmouth CollegeHanover, NH, USA; ^2^School of Sport Science, Exercise and Health, The University of Western AustraliaPerth, WA, Australia

**Keywords:** messaging, health attitudes, resistance to influence, persuasion, influence, communication theory

## Abstract

Inoculation theory, a theory of conferring resistance to persuasive influence, has established efficacy as a messaging strategy in the health domain. In fact, the earliest research on the theory in the 1960s involved health issues to build empirical support for tenets in the inoculation framework. Over the ensuing decades, scholars have further examined the effectiveness of inoculation-based messages at creating robust positive health attitudes. We overview these efforts, highlight the structure of typical inoculation-based health messages, and describe the similarities and differences between this method of counter-persuasion and other preparatory techniques commonly employed by health researchers and practitioners. Finally, we consider contexts in which inoculation-oriented health messages could be most useful, and describe how the health domain could offer a useful scaffold to study conceptual issues of the theory.

Health promotion practitioners aim to create both positive *and* resistant attitudes toward desirable health behaviors (e.g., physical activity, dietary patterns, safer sex, avoidance of harmful substances)—positive to guide healthy behavior (cf. Ajzen, 2001), and resistant to protect these positive attitudes against challenges. Indeed, healthy attitudes and behaviors are often in danger of slippage due to exposure to social, media, and peer-group factors (e.g., Prinstein and Dodge, 2008; Comasco et al., 2010), and the use of strategies to help individuals prepare for and overcome such influence is a key objective of many health promotion campaigns. Fortunately, theory, research, and anecdotal reports have provided health practitioners with assistance in their efforts to create more resistant positive health-related attitudes. One particularly strong candidate for such theory-guided efforts is *inoculation theory*—a theory that has been studied and applied in health communication, but also a theory that has, to date, not reached its fullest potential. We hope to contribute to ongoing and future work with health and inoculation theory by proposing new applied and theoretical areas for this important scholarship—work that pushes forward our understanding of persuasion *and* has applied value as a health messaging strategy to help combat serious threats to healthy living.

## Inoculation theory of resistance to influence

At the core of inoculation theory (McGuire, 1961a,b) is a biological metaphor. McGuire (1964) suggested that attitudes could be inoculated against persuasive attacks in much the same way that one's immune system can be inoculated against viral attacks. In medical immunization, weakened forms of viruses are injected into the body, and the body then reacts to this injection (e.g., through cell adaptation), protecting the body from future attacks from stronger versions of that virus. McGuire (1964) contended that by exposing individuals to a persuasive message that contains weakened arguments against an established attitude (e.g., a two-sided message, or a message that presents both counterarguments and refutations of those counterarguments), individuals would develop resistance against stronger, future persuasive attacks.

Inoculation messages involve two primary components that foster attitudinal resistance among recipients: threat and refutational preemption (but see Banas and Rains, 2010, regarding the importance of threat). *Threat* refers to recognition by message recipients that their existing position on an issue is vulnerable; it functions as a motivating force for a protective response (McGuire, 1964). One way to elicit threat (although not the only way, see Compton, 2013) is through forewarning—a direct, explicit warning that one's position on a topic is susceptible to change (McGuire and Papageorgis, 1962). The second feature of an attitudinal inoculation treatment is *refutational preemption*. This component of a message “provides specific content that receivers can employ to strengthen attitudes against subsequent change” (Pfau et al., 1997a, p. 188), and in most inoculation messages, refutational preemption is characterized by the raising and refuting of counterarguments (i.e., challenges to an existing position). Thus, a conventional inoculation message begins with a forewarning of impending challenges to a held position, then raises and refutes some possible challenges that might be raised by opponents.

For example, an inoculation message designed to discourage teen cigarette smoking (e.g., Pfau et al., 1992) might begin with a warning that peer pressure will strongly challenge their negative attitudes toward smoking, then follow this forewarning with a handful of potential counterarguments they might face from their peers (e.g., “Smoking isn't really bad for you”) followed by refutations of these counterarguments (e.g., “Actually, smoking is harmful in a number of ways…”). This inoculation format can be adapted to a number of issues, so long as (1) the intended attitude or position is already in place with message recipients, and (2) message designers are aware of some counterarguments that might be employed in attack messages in order to provide weakened, or refuted, counterarguments in the inoculation treatment message (see Ivanov, 2012, for more information on message design).

Interestingly and importantly, as part of refutational preemption, message designers do not need to raise and refute *every* potential future counterargument to be successful (Pfau, 1995). As originally argued by McGuire (1964) and confirmed by a meta-analysis of inoculation theory studies (Banas and Rains, 2010), both refutational different (i.e., where the treatment refutes challenges that do not specifically appear in a future attack) and refutational same (i.e., where the treatment refutes specific challenges that are raised) treatments confer protection, supporting the premise that inoculation messages provide “umbrella protection” against subsequent attacks. Also, encouragingly, research indicates that inoculation treatments are effective regardless of whether refutations are provided by the advertiser/messenger (i.e., “passive” refutations) or are generated by the recipient (i.e., “active” refutations; Banas and Rains, 2010). Note, too, that inoculation research has indicated a number of characteristics that make inoculation messages more effective at conferring resistance, including perceived credibility of the inoculation message source (An and Pfau, 2003) and message language that frames future attacks as threats to freedom (Miller et al., 2013).

Clearly, inoculation treatments involve dynamic, powerful processes that ultimately lead to resistance to influence. But how do these treatments differ to other commonly employed techniques among health psychologists and practitioners?

### Inoculation, implementation intentions, and stress inoculation—similarities and differences

Those well versed in health promotion techniques will likely, by this point, have considered potential overlap between components in inoculation messaging and those in implementation intention programs. Implementation intentions are designed to link anticipated situations to goal-directed responses (i.e., “If situation x arises, I will undertake y response”; Gollwitzer, 1999). A vast literature points to the benefits of these “if-then” plans for facilitating healthy behavior (e.g., Adriaanse et al., 2011; Bélanger-Gravel et al., 2013), and in some cases, the treatments look quite similar to inoculation treatments (see e.g., Rutter et al., 2006). The “if” component could very well be the counterargument mentioned in an inoculation message; the “then” component could be the refutation provided in an inoculation message. That said, methods for attitudinal inoculation differ from implementation intention programs. With attitudinal inoculation, treatments almost always contain a forewarning, specific counterarguments, and refutations of those counterarguments. In the case of implementation intention planning, however, the onus is on recipients to recognize their potential vulnerability to future challenges and to develop their own responses.

It is also worth considering the differences between treatments for attitudinal inoculation and those for stress inoculation, which are cognitive behavioral approaches to stress management (Meichenbaum and Deffenbacher, 1988). Stress inoculation involves an ongoing program between client and trainer involving three interactive phases—a conceptual educational phase, a skill acquisition and consolidation phase, and an application and follow-through phase (Meichenbaum and Deffenbacher, 1988). Although inoculation can function in interpersonal context (and recent evidence suggests that inoculation messages motivate talk about the target issue, e.g., Compton and Pfau, 2009; Ivanov et al., 2012), attitudinal inoculation can also involve brief or no interpersonal interactions, meaning that it is particularly well suited for mass campaigns or promotions. Inoculation is also suitable in situations where extended involvement with recipients is impractical or not possible.

The unique features of treatments based on inoculation theory make them ideal for use with a range of specific health problems, and they are likely to have utility beyond other commonly employed techniques used by health promotion practitioners. Some of these uses have been explored for their effectiveness already, and we detail some of these applications in the section below (see Table 1). Other health-related problems that seem ideal to treat with campaigns based on inoculation theory remain un- or under-explored.

**Table 1:** **Health-related studies on inoculation theory**.

**Health topic**	**Study**	**Participants**	**Methods**	**Primary findings**
Alcohol consumption	Godbold and Pfau, 2000	417 sixth graders (mean age = 11.2 years) from seven middle schools, USA	3 (message type: normative inoculation, informational inoculation, neutral noninoculative) × 2 (attack timing: immediate, after 2 weeks)	Exposure to the normative message was associated with significantly lower estimations of peer acceptance of alcohol use when compared to the informational and control conditions. The normative message was also superior in attitude maintenance from Time 1 to Time 3 (4 weeks later). Participants receiving an attack immediately after an inoculation message were less persuaded by the attack compared to those who had received a delay between inoculation and attack
Binge drinking	Richards and Banas, 2015	275 students at a large Mid-Atlantic university, USA	Inoculation condition compared with control; Structural equation modeling to test relationships between inoculation, threat to freedom, reactance, and intentions to drink	Inoculation messages can reduce psychological reactance
Binge drinking (and marijuana use)	Cornelis et al., 2013	156 students in three secondary schools in Belgium		Issue ambivalence moderated message effectiveness
Commercial food advertising (health- and nutrition-related claims)	Mason and Miller, 2013	145 students in Midwestern university, USA	Experiment with predictor variables of treatment/control, positive/negative outcome focus, and concrete/abstract linguistic signature of message	Inoculation messages led to resistance to deceptive health- and nutrition-related commercial food claims
Frequency of doctor visitation	McGuire, 1961a	168 students enrolled in introductory psychology course at the University of Illinois	2 (experimental condition: refutational/nonrefutational) × 2 (experimental condition: ambivalent/nonambivalent) Eight restoration conditions varying in refutational and supportive elements, and six control conditions	Issues were collapsed for analyses. No conclusions drawn about health topic
	McGuire, 1961b	168 students enrolled in introductory psychology course at the University of Illinois	Two control conditions and 4 (defense type: Active, passive, active-passive, passive-active) × 3 (attack type: no attack, same attacks, new attacks)	Issues were collapsed for analyses. No conclusions drawn about specific health topics
Legalization of marijuana	Compton and Ivanov, 2012	142 students (68 female, 74 male, Mage = 19.26 years, age range 18-27), enrolled in communication courses at a Midwestern university, USA	Three condition (control, postmessage threat assessment, interruptive threat assessment), three phase between-subjects experimental design	Participants in a non-traditional inoculation threat assessment condition (i.e., where threat was assessed after explicit forewarning but before refutational pre-emption) were more resistant to counterattitudinal attacks about marijuana legalization than participants in a control condition
	Ivanov et al., 2012	420 students from 5 universities in the USA	4 (issue: legalization of marijuana, restriction on violent television shows, banning of handguns, legalization of gambling) × 2 (experimental condition: inoculation and control) × 2 (initial attitude valance: positive, negative)	Issues were collapsed for analyses. No conclusions drawn about health topic
	Ivanov et al., 2013; study 1	101 students (71 female, 30 male), Mage = 20.93 years, age range: 19–31, enrolled in communication courses at a Southeastern university, USA	Participants received one of three inoculation messages in which certainty of attack was manipulated. Univariate ANOVA and subsequent planned comparisons assessed certainty of attack (almost certain not to occur/chance of occurrence about 50–50/almost certain to occur) as predictor variable and threat as outcome variable	Significant differences were observed between the two high certainty groups. Participants told they were very likely to face an attack to their attitudes reported greater levels of threat to those informed that an attack was unlikely
	Miller et al., 2013	420 students from five universities throughout the USA	4 (issues: legalization of marijuana, restriction on violent television shows, banning of handguns, legalization of gambling) × 3 (experimental condition: traditional inoculation, reactance enhanced, control) × 2 (counterattitudinal attack language: low controlling, high controlling)	Issues were collapsed for analyses. No conclusions drawn about health topic
	Pfau et al., 2004	443 students from introductory communication courses at University of Oklahoma, USA	Experimental design (inoculation v control), with analyses involving hierarchical multiple regression and structural equation modeling	Issues were collapsed for analyses. No conclusions drawn about health topic
	Pfau et al., 2009	281 students from introductory communication courses at a midwestern university, USA	MANCOVA, with experimental condition (control, cognitive inoculation, affective-positive inoculation, and affective-negative inoculation) as independent variable. Initial attitude toward issue position was covariate	Issues were collapsed for analyses. No conclusions drawn about health topic
	Pfau et al., 1997b	790 university students from three departments at a Midwestern university, USA	3 (experimental condition: cognitive inoculation, peripheral treatment, control) × 2 (need for cognition: lower, higher) between-subjects design	Both central and peripheral inoculation messages resulted in greater resistance to persuasive attacks against marijuana-related attitudes (relative to controls)
Mental illness (contagion of)	McGuire and Papageorgis, 1961	130 students in rhetoric courses at University of Illinois, USA	Mixed design involving 4 conditions (base belief, control, writing defense, reading defense)	Issues were collapsed for analyses. No conclusions drawn about specific health topics
Unprotected sex (and binge drinking)	Parker et al., 2012	120 university students aged 18-21 enrolled in business courses, USA	2 (experimental condition: inoculation, control) × 2 (experimental issue: unprotected sex, binge drinking) mixed experimental design	Relative to controls, inoculated participants were more able to protect their attitudes from attacks to both treated (unprotected sex) and related but untreated (binge drinking) issues
Penicillin effectiveness	McGuire, 1961a	168 students enrolled in introductory psychology course at the University of Illinois	Eight restoration conditions varying in refutational and supportive elements, and six control conditions	Issues were collapsed for analyses. No conclusions drawn about specific health topics
	McGuire, 1961b	168 students enrolled in introductory psychology course at the University of Illinois	Two control conditions and 4 (defense type: Active, passive, active-passive, passive-active) × 3 (attack type: no attack, same attacks, new attacks)	Issues were collapsed for analyses. No conclusions drawn about specific health topics
	McGuire and Papageorgis, 1961	130 students in rhetoric courses at University of Illinois, USA	Mixed design involving 4 conditions (base belief, control, writing defense, reading defense)	Issues were collapsed for analyses. No conclusions drawn about specific health topics
Smoking initiation	(a) Pfau et al., 1992	260 sixth-eighth grade students in two Northeastern USA schools	Both studies employed a 2 (experimental condition: inoculation, control) × 3 (student self-esteem: low, moderate, high) × 2 (gender: male, female) factorial design. Pfau et al. (1992) involved year 1 assessments, Pfau et al. (1992) assessed year 2 outcomes	(a) Inoculation promoted resistance to smoking initiation in year 1, but only among adolescents of low self-esteem
	(b) Pfau and Van Bockern, 1994	1047 seventh grade students in a midwestern city, USA		(b) Modest persistence for inoculative pretreatments over second year. Main effects were observed for inoculation on attitudes in the September 1991 and May 1992 assessments, in addition to an interaction effect between self-esteem and experimental condition in September 1991 that dissipated by May 1992
Teeth brushing	McGuire, 1961a	168 students enrolled in introductory psychology course at the University of Illinois	Eight restoration conditions varying in refutational and supportive elements, and six control conditions	Issues were collapsed for analyses. No conclusions drawn about specific health topics
	McGuire, 1961b	168 students enrolled in introductory psychology course at the University of Illinois	Two control conditions and 4 (defense type: Active, passive, active-passive, passive-active) × 3 (attack type: no attack, same attacks, new attacks)	Issues were collapsed for analyses. No conclusions drawn about specific health topics
	McGuire and Papageorgis, 1961	130 students in rhetoric courses at University of Illinois, USA	Mixed design involving 4 conditions (base belief, control, writing defense, reading defense)	Issues were collapsed for analyses. No conclusions drawn about specific health topics
Tuberculosis screening	McGuire, 1961a	168 students enrolled in introductory psychology course at the University of Illinois, USA	Eight restoration conditions varying in refutational and supportive elements, and six control conditions	Issues were collapsed for analyses. No conclusions drawn about specific health topics
	McGuire, 1961b	168 students enrolled in introductory psychology courses at the University of Illinois, USA	Two control conditions and 4 (defense type: Active, passive, active-passive, passive-active) × 3 (attack type: no attack, same attacks, new attacks)	Issues were collapsed for analyses. No conclusions drawn about specific health topics
Vaccination (childhood) safety (and getting an HPV vaccine)	McGuire and Papageorgis, 1961	130 students in rhetoric courses at University of Illinois, USA	Mixed design involving 4 conditions (base belief, control, writing defense, reading defense)	Issues were collapsed for analyses. No conclusions drawn about specific health topics
	Wong and Harrison, 2014	212 female students at a large Southwestern university, USA	MANCOVAs, with experimental condition as independent variable	Inoculation messages about HPV vaccination promoted resistance to messages attacking the perceived safety and efficacy toward the HPV vaccine; Inoculation messages about vaccination in general promoted resistance to messages attacking the perceived safety of HPV vaccines, but not efficacy. Inoculation also promoted childhood vaccinations, measured by attitudes and behavioral intentions

## Applications of inoculation theory in health contexts

Health is an especially robust area for inoculation research. A wide variety of issues fit within the theory's boundary conditions (e.g., the “right” attitude in place, likely attack messages from peers, media, etc.) The stakes, too, are high, affecting the well being of children and adults.

McGuire's early scholarship on inoculation theory assessed inoculation's efficacy with what he called *cultural truisms*, beliefs that were accepted without question, including the benefits of brushing one's teeth and the benefits of penicillin (McGuire, 1964). The data for these early health topics, however, were often aggregated (see Table 1), meaning that the effectiveness of the treatments on individual health issues was not focused upon. In the early stages of scholarship on inoculation theory, then, attention was directed toward theoretical advancement rather than application of the theory to health. It was not until some decades later—the 1990s—when researchers fully focused on the potential for this theory to guide health promotion. In 1992, Pfau, Van Bockern, and Kang assessed inoculation's efficacy in conferring resistance to pressures to smoke cigarettes among seventh grade students in the United States. The authors found that their inoculation treatment promoted resistance to smoking onset, but only among adolescents with low self-esteem. Later, Pfau and Van Bockern (1994) revisited the issue of adolescent smoking by following up on the student cohort that was inoculated during their earlier study. They explored whether inoculation's effects continued up to 84 weeks after the initial inoculation videos were shown. Analyses revealed no sustained impact on behavioral intentions to avoid smoking, but there was evidence of some lasting effects of the inoculation treatments on attitudes toward smoking. While acknowledging that the effects were weak, the researchers noted “the fact that the pretreatments were able to retain any influence at all during the second year is promising” (Pfau and Van Bockern, 1994, p. 425). The early findings were, indeed, good evidence for inoculation's promise as a health promotion strategy.

Over the subsequent decades, research has burgeoned on the application of inoculation theory to health-related issues. Godbold and Pfau (2000), for instance, conducted the first adolescent anti-alcohol study based on inoculation theory, providing evidence that normative-oriented inoculation messages are particularly effective in this context. More recently, Cornelis et al. (2013) and Richards and Banas (2015), have offered more empirical support for the notion that inoculation-based campaigns are effective at preserving anti-alcohol beliefs. Cornelis et al. (2013) also found that treatments associated with inoculation theory can confer resistance against pressures to engage in marijuana use, and Parker et al. (2012) discovered that inoculation messages were effective at preserving positive attitudes about condom use. Interestingly, Parker et al. also revealed that the safer sex inoculation messages promoted healthier attitudes toward risks of binge drinking—even though binge drinking was not mentioned in the inoculation message. Their findings illustrate the umbrella protection that inoculation may offer with respect to related health concerns (see also Wong and Harrison, 2014). Inoculation scholars have confirmed that inoculation can encourage adoption of healthier behaviors, including getting vaccines (e.g., Wong and Harrison, 2014), and that inoculation can lead to resistance to deceptive health- and nutrition-related claims of commercial food advertising (Mason and Miller, 2013). The promise that Pfau and his colleagues saw from their early work is being borne out in exciting, important ways in the health context. And yet, other health areas have seen little attention with inoculation research. We consider some of these next.

## Under-explored applications for inoculation-based health campaigns

Inoculation-based treatments are likely to be especially useful (relative to other techniques) in situations where specific challenges are unpredictable. After childbirth, for instance, parents often face a multitude of unforeseen challenges to their own and their baby's health, and inoculation programs could be effective at preparing parents for these challenges (e.g., prioritizing sleep, breastfeeding, nutritional choices). Inoculation also seems suitable for this context because of the speed with which messages can be delivered. Parents-to-be may have neither the time nor resources to engage in prolonged programs such as stress inoculation training; treatments based on inoculation theory can be administered extremely quickly and *prior* to the time and resource demands of early parenting.

Inoculation is also likely to be effective in areas of health in which there are common and/or powerful challenges to attitudes. In large industries (e.g., alcohol and food), marketing professionals are skilled at creating strong, creative persuasive messages that challenge attitudes (see, for example, Adams et al., 2011). Unhealthy products (e.g., food high in fat and sugar; alcohol) have been found to be over twice as commonly used in sport sponsorship as healthy products (Maher et al., 2006), and children, especially, are at a risk of being persuaded by tactics such as these (Boyland and Halford, 2013). Inoculation might help to protect against these powerful challenges that can otherwise erode established attitudes, following the early, promising results of inoculation's efficacy in the face of deceptive health- and nutrition-related claims of commercial food advertising (Mason and Miller, 2013).

## Theoretical areas for health inoculation research

Recent inoculation work has merged inoculation theory with other theories of persuasion, including psychological reactance (Miller et al., 2013). We applaud this direction and encourage continued attention to additional theories, including the Elaboration Likelihood Model (Petty and Cacioppo, 1986). Additionally, we see additional important theoretical work to be done with receiver and message variables, as outlined below, which may act as influential moderators to inoculation's efficacy in a health context.

### Receiver variables

Inoculation-based health scholarship should continue to tease out potential differences in treatment efficacy based on receiver variables. Self-efficacy, for example, affects the inoculation process with antismoking efforts (Pfau et al., 1992; Pfau and Van Bockern, 1994). Yet, other variables seem to not affect inoculation's efficacy, including gender (Pfau et al., 1992), and many variables have not yet been fully assessed in the health context, including age. Cameron (2009) posits: “Inoculation theory may be most useful for those practitioners whose patient population includes children and young adults” (p. 314); this assertion warrants empirical validation. In addition, Ivanov (2012) speculated that affect-oriented inoculation treatments (i.e., those designed to address emotions), rather than cognitively based treatments (i.e., those designed to address instrumentalities), might be more effective with younger receivers in a health context due to their more limited processing capacities. We join Ivanov and others in encouraging more work to substantiate this hypothesis and related directions, and we encourage additional exploration of non-demographic variables, such as media skepticism (Tsfati and Cappella, 2003), which could influence reception of inoculation and/or attack messages.

### Message variables

#### Treatment modality

So far, inoculation's efficacy in health behavior contexts has been assessed with print- (e.g., Parker et al., 2012) and video-based inoculation messages (e.g., Godbold and Pfau, 2000). Scholars and health practitioners do not yet have a clear understanding of how other modalities (e.g., peer interactions; social media) might influence inoculation in health contexts. Consider, for example, the efficacy of video games designed to promote better food choices and physical activity for children (Thompson et al., 2007). These video games are based on a number of theoretical constructs, including inoculation theory. The inoculation theory components appear as a “bad guy” (introducing counterarguments) and a “good guy” (introducing refutations). Although the use of video game-based inoculation treatments is at a developmental stage, this is a promising avenue for designing more enticing (and potentially, interactive) inoculation messages.

#### Attacks

The two antismoking inoculation studies (Pfau et al., 1992; Pfau and Van Bockern, 1994) did not employ conventional (i.e., standardized, external) attack messages. Rather, these investigators allowed peer pressure and other smoking influences to simply take their natural course. Future inoculation health scholarship should assess inoculation's efficacy against myriad naturally occurring attacks, including movies (e.g., Charlesworth and Glantz, 2005) and television programs (e.g., Christakis and Zimmerman, 2007). Scholars and health practitioners also need a better understanding of how inoculation fares with attacks in interpersonal contexts. More than twenty years ago, Duryea et al. (1990) noted that “Virtually no studies exist which have experimentally tested whether subjects can be successfully taught to resist nonverbal (i.e., stares, gestures) pressure to engage in health risky actions” (pp. 173–174), and a review of the literature suggests that inoculation studies still have not fully taken these interpersonal dimensions into account, although recent developments are moving in the right direction (e.g., Ivanov et al., 2012).

## Inoculating psychological constructs other than attitudes

Aside from assessing receiver variables as potential moderators to inoculation effects, more research is also encouraged to discover whether cognitions aside from attitudes can be inoculated. Attitudes are important predictors of health behavior, but so too are constructs such as self-efficacy and autonomous motivation (e.g., Biddle et al., 2007; Shields et al., 2008). These perceptions are subject to “attack” in much the same way that one's attitudes may be challenged (see e.g., Bandura, 1977; Reeve, 2009), and it is possible that these constructs can be inoculated in much the same way as attitudes. Interestingly, although Bandura (1977) outlines social conditions that facilitate or undermine self-efficacy in his social cognitive theory, and Deci and Ryan (1985) do the same for autonomous motivation in their self-determination theory, these frameworks offer few theory-based strategies for making the constructs more robust against change. The potential for inoculation theory to guide efforts at creating robust self-efficacy has recently been explored by Jackson et al. (2015); after receiving an attack to self-efficacy, participants in an efficacy inoculation condition reported greater confidence in their ability than those in a control condition. This result, which was obtained after controlling for numerous relevant variables, will hopefully encourage more inoculation scholarship on a variety of psychological constructs that are relevant for health behavior.

## Conclusions

Although inoculation has established itself as a powerful communication theory, we contend that scholars and health practitioners have not yet explored the full potential of inoculation-based health messages, despite theoretical rationale (e.g., Compton, 2013), message development guidance (Ivanov, 2012), and empirical support (e.g., Banas and Rains, 2010) to do so. What is needed next is to connect the theoretical findings of inoculation theory, in general, to the health context, in particular, and build on this work. We hope that our ideas presented here are a step in that direction. A decade ago, Compton and Pfau (2005) surmised that “It is appropriate that inoculation, inspired by a medical analogy, may make some of its most important contributions in the health context” (p. 134). Their optimism is warranted, but inoculation's full potential as a powerful health campaign strategy is yet to be realized.

## Author contributions

JC, BJ, and JD were all involved in the conception, research, and writing of this essay. Author order was determined in discussion with all authors based on significance of contributions to the overall piece.

### Conflict of interest statement

The authors declare that the research was conducted in the absence of any commercial or financial relationships that could be construed as a potential conflict of interest.
